# Prediction Models for Impending Death Using Physical Signs and Vital Signs in Noncancer Patients: A Prospective Longitudinal Observational Study

**DOI:** 10.1089/pmr.2021.0029

**Published:** 2021-10-21

**Authors:** Takahiro Hosoi, Sachiko Ozone, Jun Hamano, Kazushi Maruo, Tetsuhiro Maeno

**Affiliations:** ^1^ Department of General Medicine and Primary Care, University of Tsukuba Hospital, Tsukuba, Japan.; ^2^ Kamisu Clinical Education and Training Center, Faculty of Medicine, University of Tsukuba, Kamisu, Japan.; ^3^ Primary Care and Medical Education, Faculty of Medicine, University of Tsukuba, Tsukuba, Japan.; ^4^ Department of Biostatistics, Faculty of Medicine, University of Tsukuba, Tsukuba, Japan.

**Keywords:** impending death, noncancer patient, palliative care, prognosis

## Abstract

***Background:*** Accurate information on the prognosis in the last days of life is essential for providing better end-of-life care; however, few studies have examined the signs of impending death (SID) or developed short-term prediction models in noncancer patients.

***Objective:*** To investigate the prevalence and onset of SID and to develop models that predict death within 7 days, 72 hours, and 24 hours in noncancer patients.

***Design:*** This is a prospective longitudinal observational study.

***Setting/Subjects:*** Subjects were noncancer patients admitted to a hospital in Japan between 2019 and 2020.

***Measurements:*** We investigated 11 physical signs and vital signs every 12 hours until death after confirming a reduced daily oral intake to less than a few mouthfuls.

***Results:*** We analyzed data from 50 noncancer patients. The prediction model “pulselessness of the radial artery OR respiration of mandibular movement OR the shock Index (SI) >1.0” predicted death within 7 days with an accuracy of 83.9%, whereas the models developed to predict death within 72 and 24 hours had an accuracy of 65.0% or less. The median onset of all signs was within 3 days of death. The frequencies of decreased response to verbal stimuli and decreased response to visual stimuli were 76.0% and 74.0%, respectively.

***Conclusions:*** The prediction model using physical signs and SI predicted death within 7 days in noncancer patients with high accuracy. The prediction of death within 72 and 24 hours in noncancer patients requires investigation of physical signs not examined in this study.

## Introduction

Accurate end-of-life prognostication, particularly within seven days of death, is crucial for the provision of palliative care that increases the quality of end-of-life care for patients and their families. Previous studies reported that the behavior of bereaved families toward patients in the last days of life affected the psychological prognosis of their families.^1–4^ Information on the short-term prognosis of patients is also essential for health care professionals because of the need for important decisions that are dependent on a patient's remaining time, such as whether to perform invasive examinations and aggressive treatments or to call individuals important to the patient for last meaningful communications.^5–7^

The physical and medical changes that occur in the dying process in terminally ill cancer patients are gradually being elucidated. Various physical signs and symptoms of impending death have been investigated in these patients.^8–12^ A previous study reported that the bereaved families of cancer patients wanted doctors to provide clear information on the clinical course and symptoms in the last days of life.^13^ A diagnostic model for death within three days has been developed using symptoms, vital signs, and physical signs.^14,15^

Although end-of-life care is important in noncancer patients, few studies have investigated the dying phase in noncancer patients.^16^ Models to predict disease-specific survival outcomes in noncancer patients have been reported; however, the majority of predictions are conducted on a monthly to yearly basis.^17–19^ Limited information is currently available on the signs of impending death (SID) in noncancer patients and few short-term prognostic models have been developed. Our recent findings on terminal noncancer patients revealed that vital signs exhibited similar changes in the last seven days of life in cancer and noncancer patients.^20^ Noncancer patients may have a similar clinical course, including physical signs, as terminal cancer patients in the last days of life. Therefore, the SID in terminal cancer patients may be applied to end-of-life prognostication in noncancer patients.

The purpose of this study was to develop a prediction model of death within 7 days, within 72 hours, and within 24 hours using physical signs and vital signs in noncancer patients. We also investigated when and how often the SID appear in the last days of life in noncancer patients.

## Materials and Methods

### Study design and participants

This was a prospective longitudinal observational study. Noncancer patients (age ≥20 years) who were admitted to the general internal medicine ward of Kamisu Saiseikai Hospital in Japan between November 1, 2019, and July 30, 2020 were enrolled in this study. From April 2020, coronavirus disease 2019 (COVID-19), which is an infectious disease caused by severe acute respiratory syndrome coronavirus 2, has gradually spread in Japan as well. During the study period, this hospital accepted only a few mild cases of COVID-19 patients per month. This hospital is a regional core hospital that provides health services to a population of ∼250,000 individuals and mainly treats patients with acute illnesses. Patients who were hospitalized due to the exacerbation of noncancer diseases, not receiving artificial nutrition (tube feeding or central venous nutrition), not on a ventilator on admission, and had not been diagnosed with solid cancer with locally advanced or distant metastasis by either a pathological or clinical diagnosis were included as subjects. The institutional review board (IRB) of Kamisu Saiseikai Hospital (No. 19-0005-a) provided approval for this study. All procedures were performed in accordance with the ethical guidelines for epidemiological research presented by the Ministry of Health, Labour and Welfare of Japan and the Helsinki Declaration (as revised 2013). Since this study was a noninvasive observational study that focused on daily observations by nurses in daily clinical practice, the need for informed consent from patients was waived by the IRB based on the aforementioned ethical guidelines. Information on this study, including the purpose of using the data collected, was placed on a notice board in the hospital to provide subjects with the opportunity to opt out.

### Data collection

We reviewed previous studies^12,21,22^ to select the physical signs on which information needed to be collected in this study, and referred to the evaluation method of terminal cancer patients described by Hui et al..^10,11^ We selected the following 11 physical signs that were demonstrated to have high diagnostic characteristics for death within three days in the Investigating the Process of Dying Study^11^: a decreased response to verbal stimuli, a decreased response to visual stimuli, peripheral cyanosis, respiration with mandibular movement, death rattle, hyperextension of the neck, inability to close the eyes, drooping of the nasolabial fold, Cheyne–Stokes breathing, and pulselessness of the radial artery. Each physical sign is defined in Table 1.

**Table 1.** Definitions of Physical Signs

Physical sign	Definition	Criterion for a negative sign	Criterion for a positive sign
Decreased response to verbal stimuli	No response to a nurse's call	Absent	Present
Decreased response to visual stimuli	No response to visual stimuli (waving)	Absent	Present
Peripheral cyanosis	Bluish skin discoloration in the extremities	Absent	Present
Respiration with mandibular movement	Jaw drops during breathing	Absent	Present
Death rattle	Rattling or gurgling sound produced by air passing through airway secretions	Absent	Present
Hyperextension of the neck	Overextension of the neck	Absent	Present
Inability to close the eyes	Unable to close the eyes	Absent	Present
Drooping of the nasolabial fold	Disappearance of the nasolabial fold	Absent	Present
Cheyne–Stokes breathing	Changes in respiratory rhythm with repeated apnea and hyperpnea	Absent	Present
Pulselessness of the radial artery	Inability to palpate a pulse in the radial artery	Absent	Present
Apnea	Temporary respiratory arrest for >30 seconds	Absent	Present

After the hospitalization of patients, nurses in the internal medicine ward assessed their oral intake each day. Nurses began to systematically observe the aforementioned physical signs every ∼12 hours from the day after the confirmation of a reduced daily oral intake to less than a few mouthfuls. The appearance of a marked reduction in oral intake was adopted as the criterion for the initiation of observations because the median onset of anorexia in terminal cancer and noncancer patients was previously reported to be 7.5 and 16.5 days before death, respectively.^11,23^ Before the start of this study, a researcher (T.H.) gave a lecture on the 11 physical signs to all nurses working in the internal medicine ward. Videos and images were employed to explain these signs, and care was taken to minimize variations in evaluations among nurses. Nurses who were newly hired during the study period attended individual lectures on these physical signs. A conference was held with charge nurses at least once a month during the study period to confirm that observations were being systematically and accurately conducted. Nurses filled out a standardized data collection form, regardless of prior assessments, every 12 hours until a subject's death or the criteria described below were met. The criteria for discontinuing observations were as follows: when more than half of a meal is eaten by a subject each day for two or more days, 60 days have passed since the start of observations, the subject is discharged or transferred to another hospital, ventilator management is started, artificial nutrition is started, and the subject is diagnosed with solid cancer with locally advanced or distant metastasis by either a pathological or clinical diagnosis after the start of observations.

Age, gender, diagnosis on admission, and the presence of comorbidities categorized by the Charlson Comorbidity Index^24^ were examined as baseline characteristics. Information on vital signs, including systolic and diastolic blood pressure, heart rate, body temperature, respiratory rate, and oxygen saturation, was collected from medical records for up to seven days before death. The shock index (SI), which is useful for predicting the short-term outcome of death in terminal cancer patients,^14^ was calculated by heart rate divided by systolic blood pressure.

### Statistical analysis

Baseline characteristics were summarized using descriptive statistics. The mean value of each vital sign approximately every 12 hours from 7 days before death was plotted. We documented the frequency of each sign and the median onset from death backward using the Kaplan–Meier method. We also investigated the prevalence of each physical sign within three days before death.

The following algorithm was used to create a prediction model of death within 7 days, within 72 hours, and within 24 hours using the collected data. We examined the above 11 physical signs and SI >1.0 as predictors. Regarding each number of predictors, $m\left(m=1,\ldots ,12\right)$, and time points (within 7 days, 72 hours, and 24 hours), we searched for the best combination of *m* predictors from all 12 predictors such that the prediction probability, *P_m_*(*m* selected variables; time point), was maximized. In the case of m=2 and 7 days, for example, *P*_2_(*X*_1_, *X*_4_; 7 days) was estimated as the proportion [{*X*_1_ (e.g., “Pulselessness of the radial artery”) and/or *X*_4_ (e.g., “SI >1.0”) occurred before death} and {the subject died within 7 days of earlier of *X*_1_ and *X*_4_ events}]. We also estimated maximized prediction probabilities based on the 10-fold cross-validation method to reduce overfitting biases. We denoted raw and 10-fold cross-validation-based probabilities as Prob_raw and Prob_10fcv, respectively. Statistical analyses were performed using SPSS version 25 (IBM Japan, Ltd., Tokyo), R version 4.0.3 (R Core Team, Vienna), and Statistical Analysis System (SAS version 4.3; SAS Institute, Cary, NC).

## Results

**FIG. 1.**
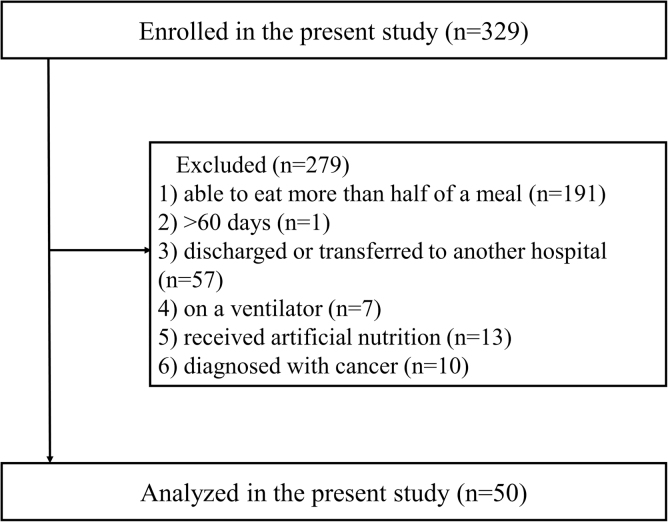
Flowchart of the study. We discontinued observations when the subject ate more than half of a meal, 60 days had passed since the start of observations, the subject was discharged or sent to another hospital, ventilator management was started, artificial nutrition was started, or the subject was diagnosed with solid cancer.

**FIG. 2.**
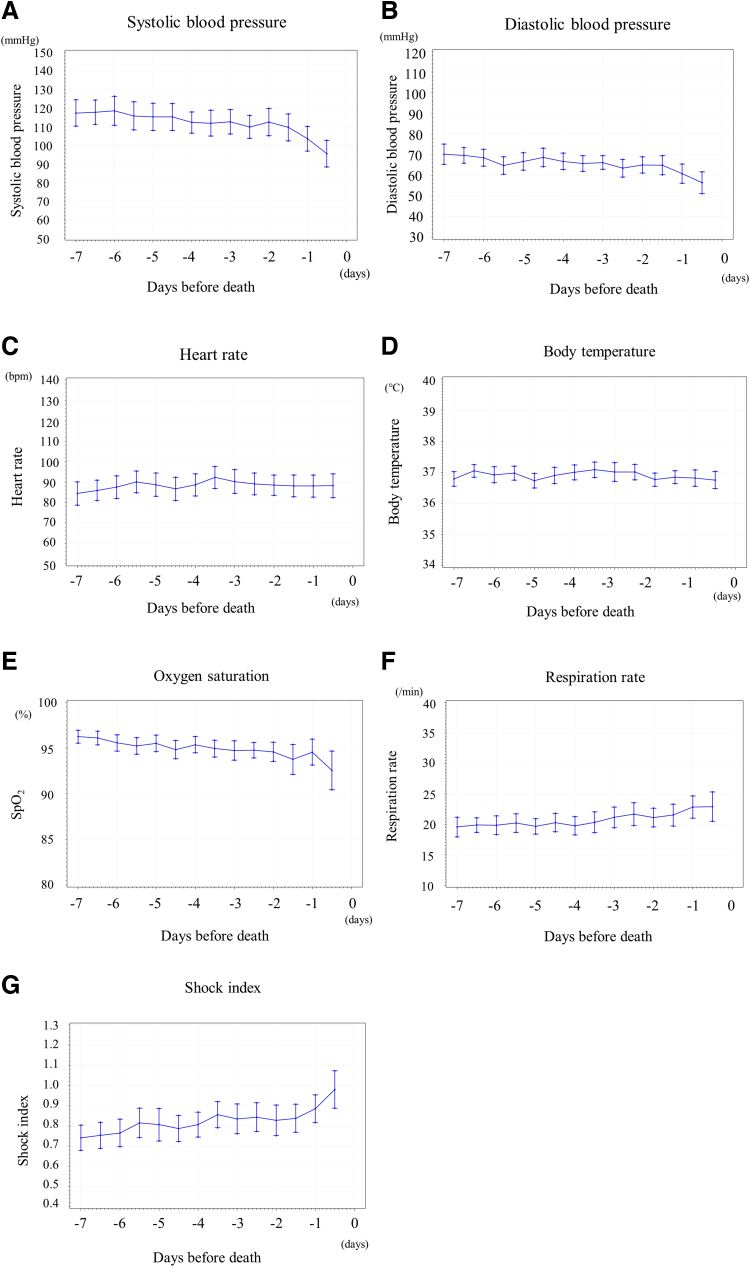
Changes in vital signs seven days before death (median ± standard deviation). **(A)** Systolic blood pressure (mmHg), **(B)** diastolic blood pressure (mmHg), **(C)** heart rate (beats/min), **(D)** body temperature (°C), **(E)** oxygen saturation, **(F)** respiration rate, and **(G)** the shock index.

In total, 329 patients were observed during the study period. After the omission of 279 patients based on our exclusion criteria, data collected from 50 patients were analyzed (Fig. 1). Table 2 shows patient characteristics. Mean age was 85.8 years and 22.9% of subjects were male. There were no COVID-19-related deaths. The average observation period and length of hospital stay were 14.1 ± 15.6 and 26.9 ± 23.2 days, respectively. Changes in vital signs and SI in the last seven days before death are shown in Figure 2.

**Table 2.** Demographics and Clinical Characteristics of Study Subjects

Baseline characteristics	All patients (*n* = 50)
Age (mean ± SD)	85.8 ± 8.2
Male (%)	16 (22.9)
Diagnosis at admission, *n* (%)	
Cerebrovascular diseases	3 (6.0)
Stroke	3
Cardiovascular disease	7 (14.0)
Chronic heart failure (ACC/AHA stage D)	7
Respiratory disease	27 (54.0)
Pneumonia	26
Chronic obstructive pulmonary disease (GOLD stage IV)	1
Liver disease	1 (2.0)
Liver cirrhosis (Child–Pugh class C)	1
Renal disease	5 (10.0)
End-stage renal disease	3
Urinary tract infection	2
Others	7 (14.0)
Dementia	4
Septic shock	3
Charlson comorbidity index (mean ± SD)	3.12 ± 1.4
Length of observation period (mean ± SD)	14.1 ± 15.6
Length of hospital stay (mean ± SD)	26.9 ± 23.2

ACC/AHA, American College of Cardiology and American Heart Association; GOLD, global initiative for chronic obstructive lung disease; SD, standard deviation.

The frequency of physical signs among patients who died during the study period is summarized in Table 3. The prevalence of a decreased response to verbal stimuli and a decreased response to visual stimuli was 76.0% and 74.0%, respectively. The prevalence of other physical signs ranged between 16.0 and 44.0% within three days of death. Apnea was not observed among the patients analyzed. The median onset of hyperextension of the neck was 3 days before death (95% confidence interval [CI]: 1.2–4.8), whereas that of Cheyne–Stokes breathing was 2.5 days before death (95% CI: 0.2–4.8). All observed physical signs had a median onset of three days or less before death. All patients with peripheral cyanosis, an inability to close the eyes, and pulselessness of the radial artery died within three days of their appearance.

**Table 3.** Prevalence, Onset, and Mortality within 72 and 24 Hours of the Appearance of Each Physical Sign

Physical sign	Median onset, days from death (95% CI)	Prevalence of the sign within 72 hours of death, *N* (%)	Mortality within 72 hours of appearance, *N* (%)	Mortality within 24 hours of appearance, *N* (%)
Decreased response to verbal stimuli	2.0 (1.5–2.5)	38 (76.0)	29/38 (76.3)	18/38 (47.3)
Decreased response to visual stimuli	2.0 (1.0–3.0)	37 (74.0)	28/37 (75.7)	16/37 (43.2)
Peripheral cyanosis	1.0 (0.4–1.6)	16 (32.0)	16/16 (100)	10/16 (62.5)
Respiration with mandibular movement	1.0 (0.7–1.3)	22 (44.0)	21/22 (95.4)	14/22 (63.6)
Death rattle	1.0 (0.8–2.5)	15 (30.0)	13/15 (86.7)	10/15 (66.7)
Hyperextension of the neck	3.0 (1.2–4.8)	14 (28.0)	8/14 (57.1)	5/14 (35.7)
Inability to close the eyes	1.0 (0.6–1.7)	11 (22.0)	11/11 (100)	8/11 (72.7)
Drooping of the nasolabial fold	1.0 (0.4–1.6)	12 (24.0)	8/12 (66.7)	7/12 (58.3)
Cheyne–Stokes breathing	2.5 (0.2–4.8)	8 (16.0)	4/8 (50.0)	2/8 (25.0)
Pulselessness of the radial artery	0.5 (0.7–1.1)	20 (40.0)	20/20 (100)	17/20 (85.0)
Apnea	None	—	—	—

CI, confidence interval.

We developed a prediction model using all 50 cases as training data. Tables 4–6 show Prob_raw within 7 days, within 72 hours, and within 24 hours when each prediction model was used and Prob_10fcv for each prediction model. Among the developed prediction models for death within seven days (Table 4), the Prob_raw of model C^7d^ (pulselessness of radial artery OR respiration with mandibular movement OR SI >1.0) was 86.0%. As a result of cross-validation, the accuracy (Prob_10fcv) of this prediction model was the highest at 83.9%. Among the developed prediction models for death within 72 hours (Table 5), the Prob_raw of model D^72h^ (pulselessness of the radial artery OR respiration with mandibular movement OR inability to close the eyes OR peripheral cyanosis) was 70.0%. Prob_10fcv of model D^72h^ was the highest at 65.0%. Among the developed prediction models for death within 24 hours (Table 6), the Prob_raw of model D^24h^ (pulselessness of the radial artery OR inability to close the eyes OR death rattle OR peripheral cyanosis) was 50.0%. The accuracy of all prediction models for death within 24 hours was <50%.

**Table 4.** Accuracy of Developed Prediction Models for Death within Seven Days

Model	Decreased response to verbal stimuli	Pulselessness of the radial artery	Shock index >1.0	Respiration with mandibular movement	Inability to close the eyes	Apnea	Death rattle	Peripheral cyanosis	Cheyne–Stokes respiration	Drooping of the nasolabial fold	Decreased response to visual stimuli	Hyperextension of the neck	Prob_raw^a^ (%)	Prob_10 fcv^b^ (%)
A^7d^	○												68.0	66.0
B^7d^		○	○										80.0	64.9
C^7d^		○	○	○									86.0	83.9
D^7d^		○	○	○	○								86.0	83.6
E^7d^		○	○	○	○	○							86.0	80.8
F^7d^		○	○	○	○	○	○						86.0	79.1
G^7d^		○	○	○	○	○	○	○					86.0	79.3
H^7d^		○	○	○	○	○	○	○	○				84.0	74.8
I^7d^	○	○	○	○	○	○	○	○		○			82.0	75.4
J^7d^	○	○	○	○	○	○	○	○		○		○	80.0	76.0

^a^ Probability that any of the variables will appear and the patient will die within seven days.

^b^ The results of a 10-fold cross-validation.

**Table 5.** Accuracy of Developed Prediction Models for Death within 72 Hours

Model	Decreased response to verbal stimuli	Pulselessness of the radial artery	Shock index >1.0	Respiration with mandibular movement	Inability to close the eyes	Apnea	Death rattle	Peripheral cyanosis	Cheyne–Stokes respiration	Drooping of the nasolabial fold	Decreased response to visual stimuli	Hyperextension of the neck	Prob_raw^a^ (%)	Prob_10fcv^b^ (%)
A^72h^	○												58.0	58.0
B^72h^	○			○									66.0	64.1
C^72h^	○			○	○								66.0	54.2
D^72h^		○		○	○			○					70.0	65.0
E^72h^		○		○	○	○		○					70.0	64.9
F^72h^		○		○	○	○	○	○					68.0	56.3
G^72h^	○	○		○	○	○		○			○		66.0	58.4
H^72h^	○	○		○	○	○		○		○	○		64.0	59.2
I^72h^	○	○		○	○	○	○	○		○	○		62.0	55.8
J^72h^	○	○		○	○	○		○	○	○	○	○	58.0	56.0

^a^ Probability that any of the variables will appear and the patient will die within 72 hours.

^b^ The results of a 10-fold cross-validation.

**Table 6.** Accuracy of Developed Prediction Models for Death within 24 Hours

Model	Decreased response to verbal stimuli	Pulselessness of the radial artery	Shock index >1.0	Respiration with mandibular movement	Inability to close the eyes	Apnea	Death rattle	Peripheral cyanosis	Cheyne–Stokes respiration	Drooping of the nasolabial fold	Decreased response to visual stimuli	Hyperextension of the neck	Prob_raw^a^ (%)	Prob_10fcv^b^ (%)
A^24h^	○												36.0	30.0
B^24h^		○			○								44.0	37.4
C^24h^		○			○		○						48.0	42.4
D^24h^		○			○		○	○					50.0	41.9
E^24h^		○			○	○	○	○					50.0	42.2
F^24h^		○			○		○	○		○		○	48.0	39.3
G^24h^		○			○	○	○	○		○		○	48.0	47.8
H^24h^		○			○	○	○	○	○	○		○	42.0	36.8
I^24h^		○		○	○	○	○	○	○	○		○	36.0	28.1
J^24h^	○	○		○	○	○	○	○		○	○	○	32.0	28.0

^a^ Probability that any of the variables will appear and the patient will die within 24 hours.

^b^ The results of a 10-fold cross-validation.

## Discussion

We herein demonstrated that a prediction model for death using physical signs and SI may predict death within seven days in noncancer patients with high accuracy. However, difficulties were associated with developing a model that predicts death within 72 and 24 hours in noncancer patients with high accuracy using the signs investigated in this study.

The accuracy of model C^7d^ (pulselessness of the radial artery OR respiration with mandibular movement OR SI >1.0) for death within seven days in noncancer patients was 83.9%. Since the area under the curve of the Objective Palliative Prognostic Score, which is a prediction model for death within seven days in terminal cancer patients, was reported to be 0.82 (95% CI: 0.75–0.89),^25^ the accuracy of our prediction model was relatively high. We consider this model to be clinically useful because it comprises signs that are relatively easy to observe, does not require special training for the evaluator, and does not take time to evaluate.

However, the accuracy of model D^72h^ (pulselessness of the radial artery OR respiration with mandibular movement OR inability to close the eyes OR peripheral cyanosis) in this study was 65.0%, which was lower than that (80%) of the algorithm to predict death within three days in terminal cancer patients reported by Hui et al..^15^ In this study, we only selected physical signs with a positive likelihood ratio of 5.0 or higher for death within three days from previous studies.^10,11^ To increase the accuracy of predicting death within 72 hours in noncancer patients, further studies that investigate physical signs not examined in this study, such as the pupillary light reflex and urine output,^11^ are needed. Moreover, the accuracy of prediction models for death within 24 hours in noncancer patients developed in this study was as low as 30–40%. It is difficult to apply this prediction model in clinical practice. A Japanese prospective observational study that examined the SID in terminal cancer patients admitted to the palliative care unit (PCU) reported that the frequency and onset of the “death rattle,” “respiration with mandibular movement,” “peripheral cyanosis,” and “pulselessness of the radial artery” were 40% (57 ± 82 hours), 95% (7.6 ± 18 hours), 80% (5.1 ± 11 hours), and 100% (2.6 ± 4.2 hours), respectively.^21^ This Japanese study also included signs that appeared transiently during the study period. More detailed observational studies may be required to more accurately predict death within 24 hours, such as increasing the frequency of observations while considering the burden on patients.

The present results also indicated that the frequency of SID differed between cancer and noncancer patients. We compared the present results with the findings of a prospective observational study on terminal cancer patients.^10,11^ The onset of physical signs in terminal cancer patients at the end of life appeared to be similar between cancer and noncancer patients. In contrast, the prevalence of physical signs other than “a decreased response to verbal stimuli,” “a decreased response to visual stimuli,” and “pulselessness of the radial artery” may differ between cancer and noncancer patients. The prevalence of “drooping of the nasolabial fold,” “inability to close the eyes,” and “apnea” in the dying phase was lower in this study than in terminal cancer patients.

Although drooping of the nasolabial fold was reported to be useful for predicting impending death in terminal cancer patients, with a prevalence of 78% within three days of death,^11^ it may not be useful in noncancer patients. The prevalence of drooping of the nasolabial fold in noncancer patients in this study was 22.2%. Since previous studies were conducted in the United States and Brazil,^10,11^ the difference in Asian facial features and the age of subjects may have influenced the results obtained. Therefore, further studies are needed to confirm whether drooping of the nasolabial fold is affected by age and race. The prevalence of an inability to close the eyes was previously reported to be 87% within three days of death in terminal cancer patients,^11^ but was 20.0% in noncancer patients in this study, and all patients who developed this sign died within three days. Although the prevalence of this sign was low in this study, it may contribute to end-of-life prognostication in noncancer patients in the dying phase. Apnea was observed within three days of death in 46% of terminal cancer patients in a previous study, but was not detected in any subjects in this study. Furthermore, Cheyne–Stokes breathing only occurred in 13.3% of noncancer patients within 72 hours of death. Although the opioid usage rate in subjects was not previously described,^10^ opioids are administered at the end of life to many cancer patients.^26^ None of the subjects in this study received opioids. The effects of opioids on the respiratory center may have contributed to the differences observed in respiratory symptoms and signs between cancer and noncancer patients in the dying phase.

There are a number of limitations that need to be addressed. First, this was a single-center study on patients admitted to the internal medicine ward of an acute care hospital in Japan. This study may have a selective bias since the rate of hospital deaths in Japan is higher than in other countries, and demographics and clinical characteristic of patients may differ in other settings. We intend to investigate the external validity of our model by conducting a multicenter study and examining other settings, such as home care and nursing homes. Second, the percentage of male subjects was low in this study. There are several possible reasons: one is that the sample size was small in this study, and the second is that, in Japan, there are more cancer deaths in men than in women. Further research is needed as it is unclear whether there is a gender difference in the prevalence of SID. Third, our subjects had acute illnesses that were mainly treated using medical therapies that may have influenced vital signs, such as antiarrhythmic drugs, steroids, and fluid infusion. However, since this study was conducted in a general internal medicine ward in Japan, the results obtained were considered to accurately reflect clinical settings in hospitals. Finally, patients were observed by nurses working in the internal medicine ward in this study; therefore, observation abilities may have differed from those of PCU nurses, who were the main observers in previous studies. Nevertheless, we considered this limitation to have had a minimal influence on the results obtained because we provided sufficient support to all nurses during the study period to standardize the evaluation.

## Conclusions

The prediction model “pulselessness of the radial artery OR respiration of mandibular movement OR SI >1.0” predicted death within seven days in noncancer patients with high accuracy. The prediction of death within 72 and 24 hours requires more detailed investigations, including the SID observed in this study.

## Abbreviations Used

AUC: area under the curve
CCI: Charlson comorbidity index
CI: confidence interval
IRB: institutional review board
PCU: palliative care unit
SI: shock index
SID: signs of impending death
